# Clinical Presentations and Outcomes of Retinoblastoma Patients in relation to the Advent of New Multimodal Treatments: A 12-Year Report from Single Tertiary Referral Institute in Thailand

**DOI:** 10.1155/2020/4231841

**Published:** 2020-09-10

**Authors:** Duangnate Rojanaporn, Taweevat Attaseth, Wimwipa Dieosuthichat, Kitikul Leelawongs, Samart Pakakasama, Usanarat Anurathapan, Ekachat Chanthanaphak, Sirintara Singhara Na Ayudhaya, Rangsima Aroonroch, Suradej Hongeng

**Affiliations:** ^1^Department of Ophthalmology, Faculty of Medicine Ramathibodi Hospital, Mahidol University, Bangkok 10400, Thailand; ^2^Department of Pediatrics, Faculty of Medicine Ramathibodi Hospital, Mahidol University, Bangkok 10400, Thailand; ^3^Department of Radiology, Faculty of Medicine Ramathibodi Hospital, Mahidol University, Bangkok 10400, Thailand; ^4^Department of Pathology, Faculty of Medicine Ramathibodi Hospital, Mahidol University, Bangkok 10400, Thailand

## Abstract

**Purpose:**

To investigate the clinical presentations and outcomes of retinoblastoma in relation to the advent of new multimodal treatments in Thailand. *Patients and Methods.* Retrospective case series. We evaluated the clinical presentation, staging, details of treatment, and treatment outcomes of retinoblastoma patients who were treated at Ramathibodi Hospital, Bangkok, Thailand, between January 1, 2007, and December 31, 2018. The log-rank test was used to explore clinical characteristics and treatment modalities that affected globe salvage and survival curves.

**Results:**

This study included 124 eyes of 81 patients with retinoblastoma. Forty-three patients (53.1%) had bilateral retinoblastoma. The median age at diagnosis was 8 months (range, 1–48 months). Of 124 eyes, 9 eyes (7.3%) had extraocular retinoblastoma and 115 eyes (92.7%) had intraocular retinoblastoma, which were classified by the International Classification of Retinoblastoma (ICRB) as group A, 4 eyes (3.5%); group B, 19 eyes (16.5%); group C, 6 eyes (5.2%); group D, 31 eyes (27%); and group E, 56 eyes (47.8%). Treatment included systemic chemotherapy, intra-arterial chemotherapy, ruthenium-106 plaque brachytherapy, external beam radiation therapy, cryotherapy, transpupillary thermotherapy, subtenon chemotherapy, and intravitreal chemotherapy. At the median follow-up period of 38.4 months (range, 0.2–148.2 months), the overall globe salvage rate of intraocular retinoblastoma was 51.7%. For unilateral retinoblastoma, globe salvage rate was 37.5% (group B, 100%; group C, 100%; group D, 50%; and group E, 18.8%). For bilateral intraocular retinoblastoma, the globe salvage rate was 57.8% (group A, 100 %; group B, 94.4%; group C, 100%; group D, 64.7%; and group E, 28.2%). The overall survival rate was 93.8%.

**Conclusions:**

Recent advanced treatment modalities have improved the probability of globe salvage. However, enucleation remains an important life-saving intervention in many advanced cases.

## 1. Introduction

Retinoblastoma is the most common primary intraocular malignant tumor in children [1], with a global incidence of approximately 7,202–8,102 annually [2]. Of these patients, approximately 40% are in Asia-Pacific countries [3]. In Thailand, the nation-wide multicenter population-based prospective study of the incidence and survival rate of childhood cancer from Thai Pediatric Oncology Group (ThaiPOG) reported 97 cases of retinoblastoma diagnosed during the year 2003–2005, placing retinoblastoma as the 7^th^ most common childhood cancer in Thailand, with the incidence of 3.1 per million population [4]. The predicted incidence of retinoblastoma in Thailand, calculated from 67 million population, is 41 children in 2013 and 2023 [3]. The overall survival probability at 5 years of retinoblastoma in Thailand from nation-wide study was 73% [4]. However, there were different treatment outcomes among each center and region of the country. Currently, there are 7 centers that provide treatment for retinoblastoma in Thailand, 4 in Bangkok (capital city), and 3 in each part of Thailand, north, northeast, and south. Previous report from 3 cancer centers in northern, northeastern, and southern Thailand during 1990 and 2009, which included 75 retinoblastoma patients, showed the survival rate of 40%, 50%, and 75%, respectively [5]. Another report from a single institute of Bangkok during 1997 to 2006, which included 90 retinoblastoma patients, showed the survival rate of 85% [6]. Numerous studies worldwide, including Asia [7–30] and Thailand [4–6, 31–34], have described the clinical manifestations and treatment outcomes of retinoblastoma, but these studies did not use IAC, brachytherapy, or intravitreal chemotherapy as part of retinoblastoma treatment [5–8, 11–13, 17–19, 23–25, 30–34]. Therefore, our study was aimed to fill in the gap in investigating clinical presentations and treatment outcomes of retinoblastoma in relation to the advent of recent treatment modalities in Thailand.

## 2. Materials and Methods

### 2.1. Patients and Study Design

This retrospective study was approved by the institutional review board of Faculty of Medicine, Ramathibodi Hospital (ID 02-60-56). We evaluated patients diagnosed with retinoblastoma between January 1, 2007, and December 31, 2018, at Ramathibodi Hospital, Bangkok, Thailand. Those with inadequate data on clinical presentation, tumor staging, and treatment modality were excluded. Demographic data included sex, ethnicity, age of onset, lag time from first presentation to diagnosis, laterality, family history of retinoblastoma, presenting signs and symptoms, follow-up time, and details of primary and adjunctive treatments were obtained from patients' medical record and analyzed statistically.

### 2.2. Staging and Treatment

Each patient underwent a complete ophthalmic examination under anesthesia, which included binocular indirect ophthalmoscopy with 360-degree indentation, Schiotz tonometer, and B-scan ultrasound. Fundus imaging and fluorescein angiography were performed using a wide-angle contact fundus camera (RetCam 3, Clarity Medical Systems, Inc., Pleasanton, CA, USA). Magnetic resonance imaging of the brain and orbits was performed in all patients to identify the presence of extraocular extension and intracranial tumors (trilateral retinoblastoma). Disease staging was classified according to International Retinoblastoma Staging System [35], and eyes with intraocular retinoblastoma were classified according to the International Classification of Retinoblastoma (ICRB) [36].

Treatment was selected based on disease stage, laterality, tumor location, visual prognosis, and input from the patients' families. For unilateral intraocular retinoblastoma, patients in ICRB groups A and B with macula- and/or papilla-sparing tumors were locally treated with cryotherapy or transpupillary thermotherapy, depending on tumor location. For ICRB group B patients with macular and/or papillary involvement and patients in ICRB group C, some patients of ICRB group D were treated with chemoreduction (CRD) or IAC. Risks and benefits of CRD and IAC were discussed in details with parents. Treatment decision was based on age of patients at the time of treatment, the feasibility of catheterization during IAC, and input from patients' families. For CRD, we followed the chemotherapy protocol from Children's Hospital of Philadelphia (CHOP) retinoblastoma [37]. The CRD regimen consisted of combined intravenous chemotherapy (carboplatin-etoposide-vincristine regimen), as well as local treatments such as cryotherapy, transpupillary thermotherapy, and plaque brachytherapy. Intravenous chemotherapy was given every 3-4 weeks for a total of six cycles. The detailed chemotherapy regimens are shown in Table 1. For IAC, we used melphalan, carboplatin, and topotecan, as described in our recent study [38]. Monthly examination under anesthesia was performed during the course of the treatment to evaluate treatment response. Primary enucleation was recommended in some unilateral ICRB group D and all unilateral ICRB group E. In case of procedure refusal, IAC or IAC combined with CRD was proposed as an alternative treatment. Each enucleated eye was examined meticulously by a histopathologist trained in the evaluation of retinoblastoma globes to identify high-risk histopathological features, including postlaminar optic nerve involvement, choroidal involvement with a diameter greater than 3 mm, anterior chamber involvement, and/or the involvement of both the optic nerve and choroid. Notably, for bilateral retinoblastoma patients, the primary treatment was CRD, with IAC reserved as an adjunctive treatment for cases of incomplete tumor response with CRD.

Intravitreal chemotherapy with melphalan or methotrexate was reserved for eyes with vitreous seeds, which poorly respond to systemic chemotherapy and/or IAC [39, 40]. Safety-enhancement procedures as previously published, including ultrasonographic biomicroscopy surveillance of the injection site, lowering of intraocular pressure by paracentesis, cryotherapy at the injection site [41], and ocular surface irrigation with sterile water [42], were performed to prevent extraocular extension of the tumor.

For patients with orbital retinoblastoma, high-dose systemic chemotherapy (carboplatin-etoposide-vincristine regimen) was administered for 3–6 cycles, followed by enucleation or exenteration, external beam radiation therapy (EBRT), and adjuvant chemotherapy, for a total of 12 cycles, as previously reported [43].

### 2.3. Statistical Analysis

Categorical variables were compared using Pearson chi-squared or Fisher's exact tests. The Kaplan–Meier method was used to estimate globe salvage and survival. The log-rank test was used to explore clinical characteristics and treatment modalities that affected globe salvage and survival curves. Univariate and multivariate Cox regression analyses were used to calculate unadjusted and adjusted hazard ratios. All statistical analyses were performed using Stata, version 15.0. *P* < 0.05 was considered statistically significant.

## 3. Results

### 3.1. Patient Characteristics

Of 86 patients, 5 were excluded from the study due to incomplete data. Thus, 81 patients (124 eyes) were included in the analysis. Forty (49.4%) patients were male. The median age at diagnosis was 8 months (range, 1–48 months). A total of 38 (46.9%) patients had unilateral disease, while 43 (53.1%) had bilateral disease. The median age of onset for bilateral disease was significantly lower than for unilateral disease (5 months vs 17 months, *P* < 0.001). The median lag time between symptom onset and visit to the oncology service was 6 weeks (range, 1–80 weeks). In our study, there were 4 patients (4.9%) with familial retinoblastoma from 3 families. Two patients were siblings from the same family, and 2 patients from 2 families had parents with retinoblastoma.

Leukocoria was the most common presenting symptom (*n* = 62 patients, 76.5%), followed by strabismus (*n* = 8 patients, 9.9%), eye screening (*n* = 4 patients, 4.9%), buphthalmos (*n* = 3 patients, 3.7%), orbital cellulitis (*n* = 2 patients, 2.5%), red eye (*n* = 1 patient, 1.2%), and poor visual behavior (*n* = 1 patient, 1.2%). Overall, 84% were treatment-naïve, while 16% were treated initially from other centers. The overall median follow-up time was 38.4 months (range, 0.2–148.2 months), which were 4.7 months (range, 0.2–9.9 months) for dead patients and 46.4 months (range, 8.4–148.2 months) for survived patients.

### 3.2. Stage

Of the 81 patients, 88.9% (*n* = 72 patients, 115 eyes) had intraocular retinoblastoma, which was unilateral in 32 (44.4%) patients and bilateral in 40 (55.6%) patients. Of 115 eyes, 4 (3.5%), 19 (16.5%), 6 (5.2%), 31 (27%), and 55 (47.8%) were classified as ICRB groups A, B, C, D, and E, respectively. For the unilateral tumor group (32 eyes, 27.8%), 1 (3.13%), 1 (3.13%), 14 (43.8%), and 16 (50%) were classified as ICRB groups B, C, D, and E, respectively. For the bilateral tumor group (83 eyes, 72.2%), 4 (4.8%), 18 (21.7%), 5 (6%), 17 (20.5%), and 39 (47%) were classified as the ICRB groups A, B, C, D, and E, respectively. Notably, the majority of eyes with intraocular retinoblastoma in our study exhibited advanced stage of disease, which was classified as ICRB groups D and E in 86 eyes (74.8%).

Extraocular extension, diagnosed on the basis of clinical and radiological manifestations, was found in 9 of 81 patients (11.1%). Of these nine patients, four (44.4%) had overt orbital disease, while 5 (55.6%) had radiologically detected extraocular extension. Lumbar puncture and bone marrow biopsy were performed in all patients with extraocular tumors. No patients had central nervous system or distant metastasis at initial presentation.

There were no statistically significant differences in median age of onset, median lag time, sex, and laterality between intraocular and extraocular tumor groups.

### 3.3. Treatment

Detailed treatment modalities of the studied eyes are shown in Tables 2 and 3. For the unilateral intraocular tumor group (32 eyes, 27.8%), 23 eyes (71.9%) were treated with globe salvage therapy. Primary enucleation was performed in 9 eyes (28.1%). Secondary enucleation was performed in 11 eyes (34.4%). For the bilateral intraocular tumor group (83 eyes, 72.2%), 69 eyes (83.1%) were treated with globe salvage therapy. Primary enucleation was performed in 14 eyes (16.9%). Secondary enucleation was performed in 21 eyes (25.3%). High-risk pathological features were found in 12 enucleated patients (21.8%), which all received postenucleation adjuvant chemotherapy. There was no local recurrence or systemic metastasis in this group of patients at a median follow-up time of 27.4 months (range, 0.4–107.7 months).

### 3.4. Treatment Outcome and Survival Outcomes

With the availability of new treatment modalities at our center, the overall globe salvage rate for the intraocular retinoblastoma group was 51.7% (groups A and C, 100%; group B, 94.7%; group D, 58%; and group E, 25%). Regarding to laterality, the globe salvage rate was 37.5% in the unilateral intraocular retinoblastoma group (group B, 100%; group C, 100%; group D, 50%; and group E, 18.8%) and 57.8% for the bilateral intraocular retinoblastoma group (group A, 100%; group B, 94.4%; group C, 100%; group D, 64.7%; and group E, 28.2%). Five patients died in our study. The overall survival rate was 93.8%. The detailed characteristics and treatment course of deceased patients are summarized in Supplement Table 1.

**Figure 1:**
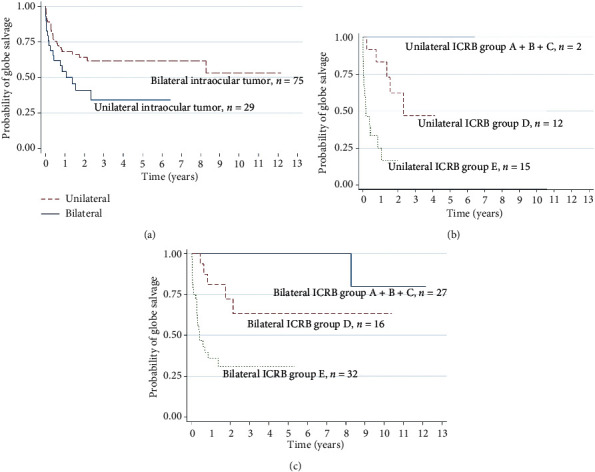
Kaplan–Meier survival analysis of globe salvage in intraocular tumors: (a) including all eyes (unilateral vs. bilateral); (b, c) unilateral and bilateral: based on the International Classification of Retinoblastoma (ICRB).

Kaplan–Meier analysis included 104 of 115 eyes with intraocular tumors, 29 eyes of unilateral intraocular tumor, and 75 eyes of bilateral intraocular tumor (Figure 1(a)); the remaining eyes were excluded because enucleation was initially performed at other center. The probabilities of globe salvage for unilateral and bilateral groups were 54% and 68% at 1 year, 41% and 64% at 2 years, and 34% and 62% at 5 years, respectively. Figures 1(b) and 1(c) show the probability of globe salvage based on ICRB classification. Kaplan–Meier analysis showed a 5-year survival rate of 93.5%.

**Figure 2:**
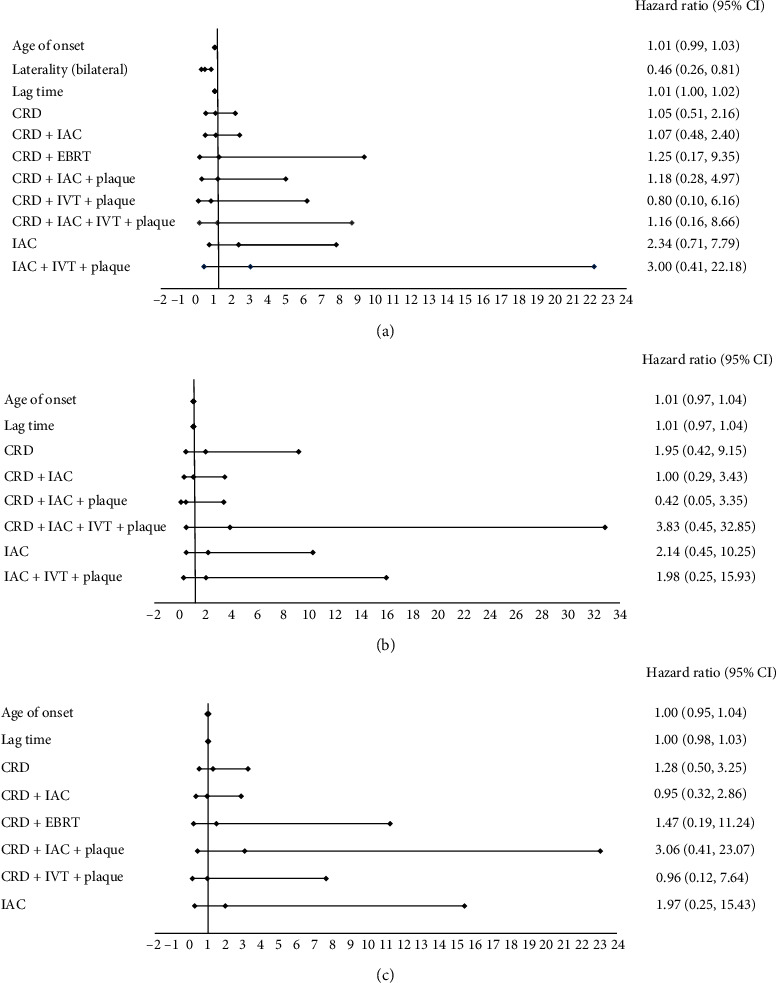
Hazard ratio of globe salvage according to patient characteristics and treatment modalities: (a) including all eyes (unilateral vs. bilateral); (b, c) unilateral and bilateral: based on the International Classification of Retinoblastoma (ICRB). CRD = chemoreduction; IAC = intra-arterial chemotherapy; EBRT = external beam radiation therapy; IVT = intravitreal chemotherapy.

The hazard ratios of globe salvage according to patient characteristics and treatment modalities are shown in Figure 2. For all eye analysis (Figure 2(a)), Cox regression analysis showed that the bilateral group had better globe salvage outcome (HR: 0.46, *P*=0.007). However, there were no statistically significant differences in globe salvage outcome with respect to other characteristics and treatment modalities by all analysis (all eyes, unilateral tumor and bilateral tumor) (Figures 2(a) and 2(c)).

## 4. Discussion

Several studies have shown that the clinical characteristics and treatment outcomes of retinoblastoma vary worldwide (Table 4). In this study, we investigated the clinical presentations and outcomes of retinoblastoma in relation to the advent of new multimodal treatments in Thailand. The median age of onset was 8 months (range, 1–48 months), which was comparable to a study from Japan (13 months) [19]. However, an older age of onset has been reported in studies from other nations, ranging from 13 to 36 months [6–8, 10, 12, 13, 17, 19, 23–25, 27, 29, 30, 45–49, 51]. Although our study showed an approximately equal proportion of unilateral and bilateral disease (46.9% and 53.1% for unilateral and bilateral disease, respectively), studies from the United States [52] and India [8] have revealed higher rates of unilateral disease than bilateral disease. Similar to other studies [6–8, 12, 13, 17, 22–25, 53, 54], the median age at diagnosis in our patients with bilateral disease was significantly lower than that of patients with unilateral disease (5 months vs 17 months, *P* < 0.001). The median lag time from first presentation until detection by an ocular oncologist was 5 weeks (range, 1–80 weeks), which was shorter than the median of 3 months reported in a study from India [13]. The shorter lag time may reflect increasing public awareness and improvements in our referral systems. No sex bias was noted in our patients, which was consistent with many previous studies [8, 54]. However, a male predominance has been reported in some studies [13], which might be attributed to the lack of attention to female children in those countries, particularly in rural areas [13]. Similar to several prior studies [5, 7–10, 12, 13, 17, 19, 23–25, 27, 29, 55], the most common presenting symptom in our study was leukocoria (76.5%), followed by strabismus (9.9%). As an initial sign, proptosis was found in 40% of cases in a study from Nepal [30], whereas none of our patients exhibited proptosis at their initial visit. Most retinoblastoma patients in developed countries exhibit intraocular tumors at presentation, while patients with extraocular tumors are rare (3%-4%) [19, 56]. In contrast, extraocular extension is commonly found in developing countries in Asia, Africa, and South America (approximately 27%–69%) [10, 13, 30, 45, 48, 49]. In our study, extraocular retinoblastoma was found in 9.8% of all cases. Our analysis showed that the median lag time was not correlated with the presence of extraocular extension. This might be related to the limited number of cases with extraocular extension in our study. Among patients with intraocular retinoblastoma, the number of patients presenting at advanced stages (ICRB groups D or E) are higher in developing countries including Thailand [6–8, 10, 12–14, 27, 29, 48]. Therefore, a major challenge in our country is the implementation of an early detection program to minimize the progression of retinoblastoma to advanced stages and allow for the treatment at earlier stages of disease and have better treatment outcomes. The main goals of retinoblastoma treatment, in descending order of importance, are to save the life, globe, and vision of the patient. In order to achieve the treatment goals, we treated our patients based on laterality, tumor staging, tumor location, visual potential, and input from patients' families.

The role of systemic chemotherapy in preventing pinealoblastoma and second neoplasm in germline retinoblastoma is controversial [57–59]. We used VEC regimen in our study for bilateral retinoblastoma patients and found that no patients in our study developed pinealoblastoma or second neoplasm during our study period. However, 5 patients died in our study, and 3 were chemotherapy-related (febrile neutropenia, chemotherapy toxicity, and chemotherapy-induced sAML). Therefore, the risks and benefits of systemic chemotherapy should be reviewed with family members before the initiation of treatment. Further studies regarding systemic chemotherapy-related complications and its role in preventing pinealoblastoma or second neoplasms are needed to identify the true risks and benefits of systemic chemotherapy in the treatment of bilateral retinoblastoma.

Because of the greater proportion of advanced retinoblastoma cases in our series, the overall globe salvage rate was 51.7%, which was much lower than that observed in developed nations [36, 44, 56]. However, globe salvage was achieved in 100% of patients in ICRB groups A and C, in 94.7% of patients in group B, in 58% of patients in group D, and in 25% of patients in group E. This was comparable to that in developed nations [36]. There was no significant association between globe salvage outcome and treatment modalities, as shown in Figure 2. This may be the result of the small sample sizes within the treatment groups.

Primary enucleation remains a life-saving intervention in advanced unilateral retinoblastoma (ICRB groups D and E) because it can effectively cure the disease, save the patients' life, reduce the risk of metastasis, reduce the number of subsequent follow-up visits, and avoid possible side effects of systemic chemotherapy or IAC. The pathological examination of enucleated eye by histopathologist trained in the evaluation of retinoblastoma globe is crucial to identify high-risk pathological features. Graduated intensity of postenucleation adjuvant chemotherapy should be given based on pathological study of each enucleated eye [60]. In Thailand and some other countries, especially in Asia, primary enucleation may be unacceptable to many patients' families. Strong advice of primary enucleation to these families can lead to treatment refusal, abandonment of the treatment, and subsequently loss of follow-up. In this situation globe salvage treatment may keep patients' families on the treatment track and make secondary enucleation more acceptable. A study from Malaysia [51] reported that most families refuse the treatment, as well as any further management, upon counseling in favor of enucleation. At our center, globe salvage treatment was offered as an alternative treatment for patients with unilateral ICRB group D or E whose families strongly refused enucleation. This was performed with the aim of preventing families from abandoning treatment altogether. All of them were informed of and accepted the risk of extraocular extension and distant metastasis. Ultimately, there was no abandonment of treatment in this group. In our study, 30 eyes had unilateral intraocular retinoblastoma group D or E. Nine eyes (30%) underwent primary enucleation, while twenty-one eyes (70%) underwent primary systemic chemoreduction and/or IAC with various adjuvant treatment modalities (Table 2). The globe salvage rate was 10 eyes (33.3%) with the median follow-up time of 26 months, and neither local recurrence nor distant metastasis was observed. However, 11 eyes (36.7%) did not respond to treatments and eventually underwent secondary enucleation as permitted by their families.

As shown in Table 4, the survival rate of retinoblastoma patients in internationally published data varies from 26% to 100%, with developed nations tending to exhibit more favorable survival outcomes than developing nations. The primary influencing factor of disease-related mortality was extraocular extension, which was generally attributed to late presentation and delay in the referral system. In our series, the overall survival rate of 93.8% was consistent with the 5-year survival estimated using Kaplan–Meier analysis. Of the five deaths recorded in our series, two were related to extraocular disease, and the remaining three were due to chemotherapy-related complications (febrile neutropenia, chemotherapy toxicity, and sAML).

The major limitation of this study was its limited scope and sample size; all data were obtained from a single referral center in Bangkok, which may not entirely represent retinoblastoma patients in Thailand. A multicenter national study and a national system of retinoblastoma registration are required to establish a nation-wide picture of this disease. Education of the public and primary health care providers, along with the implementation of a retinoblastoma screening program, should be promoted to enable earlier detection of retinoblastoma. This would enable patients to achieve better treatment outcomes. In addition, proper retinoblastoma management requires multidisciplinary cooperation, which includes medical personals, nurses, ocularists, ocular oncologists, pediatric oncologists, interventional neuroradiologists, neurosurgeons, anesthesiologists, and ocular pathologists. Because these specialized providers are not available in many areas of the country, comprehensive referral centers may provide a solution for the enhanced provision of retinoblastoma care in Thailand.

## 5. Conclusions

In summary, we have shown the clinical presentation and treatment outcomes of retinoblastoma patients who underwent recent multimodal treatments at our center, spanning a period of 12 years. Although the treatment of retinoblastoma remains a complex challenge in Thailand, the recent availability of advanced treatment modalities at our center has enhanced our capacity to save lives and has increased the likelihood of globe salvage. The current study fills a gap in the existing literature by reporting clinical presentations and outcomes of retinoblastoma with the recent multimodal treatments in Thailand.

## Figures and Tables

**Table 1:** Retinoblastoma chemotherapy regimens [37, 43].

Agents/course interval	Regimens		
	Standard dose (BW < 12 kg)	Intensified dose for ICRB groups D and E (BW < 12 kg)	High dose for extraocular retinoblastoma
Vincristine	1.5 mg/m^2^/day on day 1 (0.05 mg/kg/day)	1.5 mg/m^2^/day on day 1 (0.05 mg/kg/day)	0.025 mg/kg/day on day 1

Etoposide	150 mg/m^2^/day on days 1-2 (5 mg/kg/day)	180 mg/m^2^/day on days 1-2 (6 mg/kg/day)	12 mg/kg/day on days 1-2

Carboplatin	560 mg/m^2^/day on day 1 (18.6 mg/kg/day)	420 mg/m^2^/day on days 1-2 (14 mg/kg/day)	28 mg/kg/day on day 1

Course interval	Continue every 4 weeks for a total of six cycles	Continue every 4 weeks for a total of six cycles	Continue every 3 weeks for a total of six cycles

ICRB = International Classification of Retinoblastoma.

**Table 2:** Treatment modalities and globe salvage rates for intraocular tumor according to International Classification of Retinoblastoma (ICRB).

Treatment modalities, *n* = 115	ICRB group, eyes (globe salvage, %)									
	A		B		C		D		E	
	Unilateral	Bilateral	Unilateral	Bilateral	Unilateral	Bilateral	Unilateral	Bilateral	Unilateral	Bilateral
CRD	—	4 (100%)	1 (100%)	9 (100%)	—	2 (100%)	—	9 (66.7%)	2 (0%)	19 (47.4%)
CRD + plaque	—	—	—	2 (100%)	1 (100%)	—	—	—	—	
CRD + EBRT	—	—	—	1 (100%)	—	—	—	1 (0%)	—	
CRD + IAC	—	—	—	2 (100%)	—	1 (100%)	4 (75%)	4 (100%)	3 (0%)	6 (33.3%)
CRD + IAC + IVT	—	—	—	1 (100%)	—	2 (100%)	1 (100%)	—	—	
CRD + IAC + plaque	—	—	—	—	—	—	4 (75%)	1 (0%)	—	
CRD + IVT + plaque	—	—	—	2 (50%)	—	—	—	—	—	
CRD + IAC + IVT + plaque	—	—	—	1 (100%)	—	—	1 (0%)	—	—	
IAC	—	—	—	—	—	—	1 (0%)	1 (100%)	3 (67.7%)	1 (0%)
IAC + IVT	—	—	—	—	—	—	—	—	1 (100%)	
IAC + IVT + plaque	—	—	—	—	—	—	1 (0%)	—		
Primary enucleation (in our center/from referral hospital)	—	—	—	—	—	—	2 (1/1)	1 (0/1)	7 (7/0)	13 (8/5)
Secondary enucleation (in our center/from referral hospital)	—	—	—	1	—	—	5 (5/0)	5 (5/0)	6 (6/0)	15 (14/1)

Total	0	4 (100%)	1 (100%)	18 (94.4%)	1 (100%)	5 (100%)	14 (50%)	17 (64.7%)	16 (18.8%)	39 (28.2%)

ICRB = International Classification of Retinoblastoma; CRD = chemoreduction; IAC = intra-arterial chemotherapy; EBRT = external beam radiation therapy; IVT = intravitreal chemotherapy.

**Table 3:** Treatment modalities for intraocular and extraocular tumors according to the International Retinoblastoma Staging System (IRSS).

Treatment modalities, *n* = 124	IRSS staging, eyes									
	0		I		II		III		IV	
	Unilateral	Bilateral	Unilateral	Bilateral	Unilateral	Bilateral	Unilateral	Bilateral	Unilateral	Bilateral
Globe salvage treatment^1^	12	48	—	—	—	—	—	—	—	—
CRD + enucleation	—	—	3	15	—	—	—	—	—	—
CRD + IAC + enucleation	—	—	4	4	—	—	—	—	—	—
CRD + EBRT + enucleation	—	—	—	1	—	—	1	—	1	—
CRD + IAC + plaque + enucleation	—	—	1	1	—	—	—	—	—	—
CRD + IAC + IVT + plaque + enucleation	—	—	1	—	—	—	—	—	—	—
CRD + IVT + plaque + enucleation	—	—	—	1	—	—	—	—	—	—
CRD + exenteration	—	—	2	—	—	—	—	—	—	—
CRD + exenteration + EBRT	—	—	—	—	—	—	1	—	—	—
IAC + enucleation	—	—	2	1	—	—	—	—	—	—
IAC + IVT + plaque + enucleation	—	—	1	—	—	—	—	—	—	—
Primary enucleation (in our center/from referral hospital)	—	—	9 (8/1)	14 (8/6)	—	—	—	—	—	1 (0/1)

Total	12	48	23	37	0	0	2	0	1	1

^1^Detailed treatment modalities are listed in Table 1. IRSS = International Retinoblastoma Staging System; CRD = chemoreduction; IAC = intra-arterial chemotherapy; EBRT = external beam radiation therapy; IVT = intravitreal chemotherapy.

**Table 4:** Summary of retinoblastoma patient characteristics and treatment outcomes in the published literature^1^.

Author	Year of publication	Country	No. of patients/eyes	Median/mean age of onset (months)	Most common presenting symptom (%)	% eyes with advanced ICRB groups D and E	% patients with EOE	Patient survival rate	Globe salvage rate for intraocular tumor	Follow-up time (median or mean)
Our study	2018	Thailand	81/124	8 (1–48)	Leukocoria (76.5%)	70.2%	6.5%	93.8%	51.7%	38 (0.2–148) months
Francis et al. [44]	2018	USA	46/92	5.48 (median) (0.33–27.9)	NA	48.9%	6.52%	97.83%	91.30%	38.7 (6.9–60.9) months
Goolam et al. [45]	2018	South Africa	245/330	32.6 (mean) ± 26.8	Leukocoria (37%)	19% IRSS stage 0-1	69.39%	58%	NA	71.9 (3–238) months
Kaliki et al. [8]	2017	India	1457/2074	24 (<1–370)	Leukocoria (75%)	73%	9% (eye)	92%	45% for all eyes	30 (3–234) months
Al Hasan et al. [9]	2017	Syria	37/65	Age 4 months to 1 year in 40.6%	Leukocoria (57%)	NA	13.50%	NA	NA	NA
Soliman et al. [7]	2017	Egypt	47/70	24 (2–49)	Leukocoria (96%)	64%	0%	100%	NA	NA
Berry et al. [46]	2017	USA	294/345	19.8 (mean)	NA	Group D 47.8%; group E 52.2%	0.9% (patient)	98.5	40.3%	86.3 (mean) ± 62.5 months
Li et al. [11]	2016	Taiwan	154/NA	NA	NA	NA	NA	83%	NA	NA
Gao et al. [12]	2016	China	253/303	21 (0–611)	Leukocoria (71%)	90%	9%	91%	21.70%	16 (0.3–119) months
Selistre et al. [10]	2016	Brazil	140/187	23.5 (mean)	Leukocoria (74%)	78% of all eyes	36.40%	86%	29.30%	323 (300–346) months
Chawla et al. [13]	2016	India	600/794	21 (1–150)	Leukocoria (83%)	78%	27.70%	76%	28.2	21 (1–60) months
Lumbroso-Le Rouic et al. [15]	2015	France	730/1049	NA	NA	NA	NA	98.5	NA	93 (median follow-up time) months
Gichico et al. [16]	2015	Kenya	160	NA	NA	NA	NA	27%	NA	37.5 (1–144) months
Waddell et al. [14]	2015	Uganda	270/NA (181 before introduction of chemotherapy, 89 after introduction of chemotherapy)	NA	NA	98% after introduction of chemotherapy had group E or EOE	NA	45% (before introduction of chemotherapy) and 65% (after introduction of chemotherapy)	NA	1–86 months
Okimoto et al. [19]	2014	Japan	34/43	13 (0.7–74)	Leukocoria (97%)	86%	2.90%	97%	25.60%	106.6 ± 53 months
Park et al. [18]	2014	Korea	600/NA	NA	NA	NA	NA	92%	NA	10 years
da Rocha-Bastos et al. [22]	2014	Portugal	46/59	Mean age: 22.19 for unilateral, 6.92 for bilateral	Leukocoria (37%)	54% Reese–Ellsworth IV-V	NA	98%	23.70%	12 (1–33) years
Moreno et al. [20]	2014	Argentina	438/577	Unilateral: 35 for lower EHDI and 24 for higher EHDI. Bilateral: 11.5 for lower EHDI and 9 for higher EHDI	NA	NA	NA	89%	NA	NA
Subramaniam et al. [17]	2014	Malaysia	119/162	22 (1–123)	Leukocoria (92%)	NA	NA	55%	NA	1 year
Al-Nawaiseh I et al. [47]	2014	Jordan	71/114	21.7 (mean) (1–276)	Leukocoria (54%)	48% Reese–Ellsworth I–IV; 52% Reese–Ellsworth V	NA	NA	63.2%	26.9 (0.25–160) months
Lim et al. [23]	2013	Singapore	51/67	25.7 (SD 19.9)	Leukocoria (71%)	88%	0%	91%	23.90%	5 years
Nabie et al. [24]	2012	Iran	40/57	20 (2 weeks–10.2 years)	Leukocoria (98%)	NA	NA	NA	26.3% for all eyes	NA
Luna-Fineman S et al. [48]	2012	Central America	171/213	28 (median) (1–108)	NA	80% Reese–Ellsworth IV–V	62%	52%	NA	18 (median) (1–112) months
Ali AAE et al. [49]	2011	Sudan	25/31	36 (median) (8–60)	Enlarged eye (56%)	NA	64%	56%	NA	15.4 (median) (9–36) months
Essuman et al. [27]	2010	Ghana	23/27	24.0 ± 11.3	Leukocoria (87%)	87% Reese–Ellsworth V	17%	26%	NA	5.7 months
Zhao et al. [25]	2010	China	470/595	20 (2 weeks–10.2 years)	Leukocoria (73%)	NA	NA	NA	NA	NA
Atchaneeyasakul et al. [6]	2009	Thailand	90/116	18 (0.5–80)	NA	76%	14%	85%	16% for all eyes	27 months
Broaddus et al. [50]	2009	USA	992/NA	NA	NA	NA	NA	95%	NA	5 years
MacCarthy et al. [28]	2008	Great Britain	1576/2156	NA	NA	NA	NA	Unilateral cases diagnosed: 1963–1967, 85%; 1998–2002, 97%. Bilateral cases diagnosed: 1963–1967, 88%; 1998–2002, 100%	NA	5 years
Ozdemir et al. [29]	2007	Turkey	91/121	18 (2–100)	Leukocoria (65%)	79% Reese–Ellsworth IV-V	20.9%	92%	46.3% for all eyes	35 (4–63) months
Shields et al. [36]	2006	USA	163/249	NA	NA	44%	0	100%	73.10%	6.2 (1–10.6) years
Badhu et al. [30]	2005	Nepal	43/47	3.04 ± 1.80 years	Proptosis (40%)	NA	46.5%	53.5%	NA	Minimum 2 years

^1^Search strategy: scientific literatures on retinoblastoma treatments written in English published between 2005 and 2020 were searched using PubMed. The search terms included retinoblastoma, retinoma, retinocytoma, chemoreduction, intra-arterial chemotherapy, brachytherapy, external beam radiation therapy, intravitreal chemotherapy, enucleation, exenteration, globe salvage rate, and survival rate. The literatures from each countries/regions were selected based on well-documented treatment modalities, tumor staging, and treatment outcomes. ICRB = International Classification of Retinoblastoma; EOE = extraocular extension; NA = nonapplicable; EHDI = Extended Human Development Index.

## Data Availability

All the data used to support the findings of this study are included within the article and are available from the corresponding author by a reasonable request.

## Supplementary Materials

Supplementary Materials Supplement Table 1. The detailed characteristics and treatment course of dead patients.
