# Supplementary material for: Clinical Presentations and Outcomes of Retinoblastoma Patients in relation to the Advent of New Multimodal Treatments: A 12-Year Report from Single Tertiary Referral Institute in Thailand
Source: J Ophthalmol. 2020 Sep 10;2020:4231841. doi: 10.1155/2020/4231841 (PMC7508219; doi:10.1155/2020/4231841)
Supplement: Supplementary Materials — Supplement Table 1. The detailed characteristics and treatment course of dead patients. [file 4231841.f1.docx]

Supplement table 1. The detailed characteristics and treatment course of dead patients.

| Patient | Sex (M/F) | Laterality | Familial RB | ICRB group/EOE | Presenting signs/symptoms | Age of onset (months) | Lag time (weeks) | Age of death (months) | Primary treatment received | Secondary treatment received | Pathology result | Cause of death |
| --- | --- | --- | --- | --- | --- | --- | --- | --- | --- | --- | --- | --- |
| 1 | M | Unilateral | No | EOE | Orbital cellulitis | 3 | 2 | 13 | Intravenous ceftriaxone (good response) and enucleation following recurrent orbital cellulitis | Systemic chemotherapy and EBRT | Diffuse infiltrating retinoblastoma. Massive choroidal involvement with scleral invasion. Tumor at optic nerve cut end. | Brain metastasis |
| 2 | M | Bilateral | No | Bilateral group E | Leukocoria | 2 | 7 | 8 | Systemic chemotherapy 6 cycles (intensified dose for ICRB group D and E) | Subtenon carboplatin both eyes | NA | Febrile neutropenia 2 weeks after the last cycle of chemotherapy |
| 3 | F | Bilateral | No | Bilateral group B | Incidental finding during ROP screening | 2 | 0 | 3 | Systemic chemotherapy | None | NA | Chemotherapy overdose (other center) |
| 4 | F | Unilateral | No | Group D | Leukocoria | 2 | NA | 44 | Enucleation | Post enucleation systemic chemotherapy 6 cycles | Massive choroidal and postlaminar involvement.  Free surgical margin | Chemotherapy-induced sAML. The latency period between the first dose of chemoprophylaxis and development of sAML was 21 months |
| 5 | M | Bilateral | Yes | Group B OD and EOE OS | Leukocoria | 20 | 52 | 44 | Primary enucleation OS,  systemic chemotherapy, cryotherapy OD (other center)  No EBRT | RU-106 OD | Tumor at optic nerve cut end | Brain metastasis |

EOE = Extraocula extension; EBRT = External beam radiation therapy; ICRB = International Classification of Retinoblastoma; ROP = Retinopathy of prematurity; sAML = Secondary acute myelogenous leukemia; RU-106 = Ruthenium-106
